# Short-term changes in interleukin 10 following drug-eluting stent placement in stable angina patients: Influence of hypertension

**DOI:** 10.21542/gcsp.2026.2

**Published:** 2026-02-28

**Authors:** Zeyad Albadri, Monte Badri, Reem Almousali

**Affiliations:** 1 Department of Clinical and Experimental Medicine, Faculty of Health Sciences, Linköping University, Linköping, Sweden; 2 Department of Medicine, Ljungby General Hospital, Sweden; 3 Medical Department, Bra Liv Medical Centres, Huskvarna, Sweden

## Abstract

**Background:** Drug-eluting stents (DES) are widely used in percutaneous coronary interventions (PCI) to reduce restenosis. However, their effect on acute inflammatory and anti-inflammatory responses is not completely understood. Interleukin-10 (IL-10) is a key anti-inflammatory cytokine implicated in vascular healing and modulation of immune responses following endothelial injury.

**Objective:** To evaluate the acute-phase response of IL-10 following DES implantation in patients with stable angina, with a particular focus on differences between hypertensive and normotensive individuals.

**Methods:** Thirteen patients with stable angina undergoing elective DES implantation were included. Arterial blood samples were collected at baseline and immediately post-procedure, and venous samples were collected at 24 h post-PCI. Serum IL-10 levels were measured using ELISA. Patients were stratified based on hypertensive status.

**Results:** IL-10 levels significantly increased immediately after stenting (16.65 ± 1.49 pg/ml) compared to baseline (11.88 ± 0.42 pg/ml; *P* < 0.05). However, levels declined significantly at 24 h post-procedure (10.15 ± 1.04 pg/ml). Hypertensive patients showed a greater post-procedure increase in IL-10 (18.16 ± 2.4 pg/ml) than normotensive patients (15.16 ± 1.08 pg/ml), though this difference was not statistically significant. At 24 h, IL-10 levels fell below baseline in both groups. The IL-6/IL-10 ratio, used as an index of net inflammatory activity, significantly increased at 24 h (1.05 ± 0.13), suggesting a delayed shift toward a pro-inflammatory state.

**Conclusion**: DES implantation in patients with stable angina induces a transient increase in IL-10 levels, particularly among hypertensive individuals. The subsequent decline in IL-10 and rise in the IL-6/IL-10 ratio at 24 h may indicate a pro-inflammatory rebound phase. These findings underscore the dynamic nature of cytokine regulation following PCI and support further investigation into IL-10’s role in modulating vascular inflammation and restenosis risk.

## Background

Percutaneous coronary intervention (PCI) with drug-eluting stent (DES) implantation has become a cornerstone in the treatment of stable coronary artery disease. Compared to bare-metal stents (BMS), DES significantly reduce neointimal hyperplasia and restenosis due to the localized delivery of antiproliferative drugs such as sirolimus or everolimus^1^. Despite these benefits, stent deployment inherently causes mechanical vascular injury, which initiates an acute inflammatory response that can affect endothelial repair and long-term vascular outcomes^2^.

Among the cytokines involved in this response, interleukin-10 (IL-10) plays a key anti-inflammatory and atheroprotective role. It acts by inhibiting the production of pro-inflammatory cytokines, matrix metalloproteinases, and adhesion molecules, and by downregulating NF-*κ*B signaling pathways^3^. In patients undergoing PCI, IL-10 has been identified as a relevant biomarker of vascular immune response, with elevated levels shortly after stent implantation, suggesting a compensatory anti-inflammatory effect^4,5^.

Recent clinical data suggest that this elevation in IL-10 is short-lived, typically peaking shortly after PCI and declining to baseline or lower levels within 24 to 48 h post-procedure^5,6^. This transient response implies that IL-10 expression is tightly regulated following endothelial injury. Notably, in elderly patients, a diminished IL-10 response has been associated with a higher risk of in-stent restenosis, highlighting its potential prognostic value^5–7^.

Hypertension, which commonly coexists with coronary artery disease, is independently linked to chronic systemic inflammation and elevated levels of pro-inflammatory cytokines like IL-6^8^. However, its influence on IL-10 levels post-PCI remains inadequately characterized. Emerging evidence suggests that hypertensive individuals may exhibit altered cytokine dynamics, potentially affecting the delicate balance between pro- and anti-inflammatory responses after stent implantation^8^.

This study seeks to define the temporal profile of IL-10 following DES implantation in patients with stable angina and to explore the potential modulatory role of hypertension in this anti-inflammatory response.

## Methods and materials

### Study population and design

This was a prospective observational study. Thirteen patients (8 males, 5 females; mean age 62 ± 4.1 years) with documented stable angina pectoris undergoing elective PCI with DES implantation were enrolled after obtaining informed written consent.

Patients with acute myocardial infarction, cardiogenic shock, unstable angina, or known intolerance to aspirin, heparin, or clopidogrel were excluded. Subjects with hypertension were included only if blood pressure was in the mild to moderate range (systolic 140–179 mmHg or diastolic 90–109 mmHg), in accordance with the British Hypertension Society classification, and controlled on stable antihypertensive therapy.

Given the limited number of included patients, this study should be regarded as a pilot, hypothesis-generating investigation. The sample size was not powered to detect small or moderate differences between subgroups, particularly in analyses stratified by hypertensive status. Consequently, the findings are intended to describe temporal cytokine patterns following drug-eluting stent implantation and to inform the design of larger, adequately powered studies rather than to provide confirmatory evidence.

### Procedure

All patients received a standard pre-treatment with aspirin (75 mg daily) and clopidogrel (75 mg daily). PCI was performed via femoral access under local anesthesia using a 7 French sheath for arterial cannulation. Heparin (5000 units IV) was administered prior to the procedure and continued post-operatively to maintain an activated partial thromboplastin time (aPTT) of 1.5–2 × baseline. Coronary lesions were treated using a monorail balloon catheter system with pre- and post-dilation as indicated. Each patient received a single drug-eluting stent sufficient to cover the target lesion.

### Blood sampling

Blood samples were obtained at three time points. At baseline immediately before balloon inflation from the femoral artery sheath, post PCI procedure within 10 min after stent deployment from the femoral artery and 24 h post-procedure via venous puncture from the brachial vein. Samples were collected in heparinized tubes and centrifuged at 1600 g for 15 min. Plasma was aliquoted and stored at −80 °C until analysis.

Arterial blood sampling at baseline and immediately post-procedure was performed via the femoral sheath to allow sampling during PCI without additional vascular puncture. Venous sampling at 24 h was performed via peripheral venepuncture in accordance with routine post-procedural care.

### Cytokine measurement (IL-10 and IL-6)

The IL-6/IL-10 ratio was evaluated as an exploratory, post-hoc index of the balance between pro- and anti-inflammatory activity and was not predefined as a primary or secondary outcome. Plasma concentrations of IL-10 and IL-6 were determined using a commercially available sandwich ELISA (enzyme-linked immunosorbent assay) following the manufacturer’s instructions (Biosource, Camarillo, USA for IL-10; R&D Systems, Minneapolis, USA for IL-6). All measurements were performed in triplicate, and a standard curve was generated using serial dilutions of recombinant human cytokines. Optical density was measured at 450 nm using a microplate reader (Labsystems Multiskan RC, UK), and results were expressed in pg/ml.

### Statistical analysis

Continuous variables are presented as mean ± standard error of the mean (SEM). Within-subject comparisons of cytokine levels across time points (baseline, immediate post-procedure, and 24 h) were performed using repeated-measures analysis of variance or the Wilcoxon signed-rank test, as appropriate based on data distribution. Between-group comparisons (hypertensive vs. normotensive patients) were conducted using the Mann–Whitney U test or unpaired Student’s *t*-test, as appropriate. The IL-6/IL-10 ratio was analyzed descriptively as an exploratory marker of net inflammatory balance. Given the exploratory and pilot nature of the study, no formal correction for multiple testing was applied; this limitation is acknowledged in the discussion. A two-sided *p*-value <0.05 was considered statistically significant.

### Ethical approval

Ethical approval for the study was granted by the Research Ethics Committee approval number 2503/030303. The study was conducted in accordance with the principles outlined in the Declaration of Helsinki. Written informed consent was obtained from all participants prior to inclusion. As this was a small prospective observational study involving standard of care procedures without randomization or experimental intervention, it was not registered in a clinical trial database.

## Results

### Patient characteristics

A total of 13 patients with stable angina were included in the study. The mean age was 62 ± 4.1 years. Of the participants, 8 were male and 5 female. Five patients (38%) had documented mild-to-moderate hypertension and were on antihypertensive therapy. Other baseline characteristics included diabetes mellitus (n = 4), hyperlipidemia (n = 5), and current smoking (n = 6). All patients received aspirin, and the majority were also treated with beta-blockers (n = 9), nitrates (n = 12), ACE inhibitors (n = 10), and statins (n = 6) (Table 1).

### Changes in IL-10 levels in total cohort

**Figure 1.**
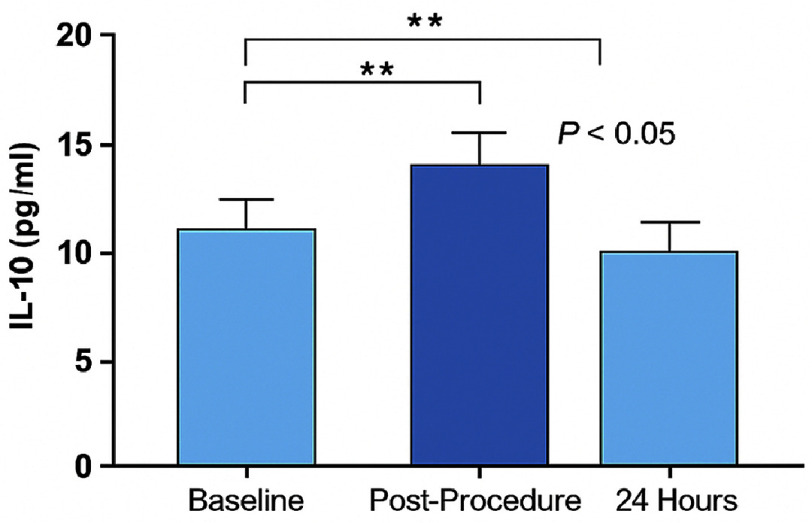
Changes in IL-10 levels in the total cohort. Mean ± SEM IL-10 concentrations are shown at Baseline, Post-Procedure, and 24 Hours after stent deployment. Asterisks indicate statistically significant differences (*P* < 0.05).

Serum IL-10 concentrations increased significantly immediately after drug-eluting stent implantation, rising from a baseline value of 11.88 ± 0.42 pg/ml to 16.65 ± 1.49 pg/ml (*P* < 0.05). By 24 h post-procedure, IL-10 levels declined significantly to 10.15 ± 1.04 pg/ml, falling slightly below the initial baseline values (*P* < 0.05 compared to post-procedure) (Figure 1).

**Figure 2.**
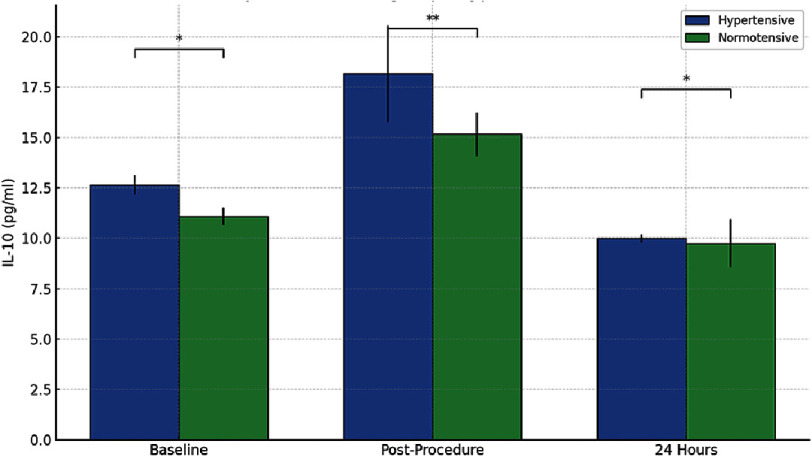
IL-10 levels in hypertensive vs normotensive patients over time. Serum IL-10 levels at baseline, immediately after drug-eluting stent implantation, and 24 h post-procedure in hypertensive and normotensive patient subgroups. Both groups showed an acute IL-10 elevation post-stenting followed by a decline at 24 h, with hypertensive patients exhibiting a slightly greater initial increase. Data are shown as mean ± SEM. *P* < 0.05 (*), *P* < 0.01 (**).

**Table 1:** Baseline characteristics of the patients.

Patients Characteristics	
No. of patients	13
Age, years	62 ± 4.1
Male gender, n (%)	8 (61%)
Diabetes, n (%)	4 (31% )
Hypertension, n (%)	5 (38%)
Dyslipidaemia, n (%)	6 (46%)
Instable Angina, n (%)	13 (100%)
Medications	
Statins, n (%)	6 (46%)
Beta-blockers, n (%)	9 (69%)
ACEIs/ARB, n (%)	10 (77%)
Nitrates, n (%)	12 (92%)
Smoking, n (%)	6 (46%)

**Notes.**

ACEI angiotensin-converting enzyme inhibitors ARB angiotensin receptor blockers

### IL-10 dynamics in hypertensive and normotensive subgroups

In hypertensive patients (n = 5), IL-10 increased from 12.66 ± 0.49 pg/ml at baseline to 18.16 ± 2.4 pg/ml post-procedure, representing a 44% increase. However, levels dropped significantly to 10.00 ± 0.19 pg/ml at 24 h, below baseline values (*P* < 0.01).

In normotensive patients (n = 8), IL-10 rose from 11.09 ± 0.43 pg/ml to 15.16 ± 1.08 pg/ml post-procedure and declined to 9.75 ± 1.19 pg/ml at 24 h. While both groups demonstrated a similar trend, hypertensive patients exhibited a slightly greater magnitude of IL-10 elevation post-procedure, though the difference between groups was not statistically significant at any time point (Figure 2).

### IL-6/IL-10 ratio

An exploratory analysis of the IL-6/IL-10 ratio was performed as an approximate index of net inflammatory activity. The ratio slightly decreased post-procedure from 0.41 ± 0.04 at baseline to 0.39 ± 0.03. However, a significant increase was observed 24 h after stenting, reaching 1.05 ± 0.13 (*P* < 0.01 vs. baseline and post-procedure). This suggests a delayed shift toward a pro-inflammatory profile in the post-stenting period.

## Discussion

This study aimed to evaluate the acute-phase response of the anti-inflammatory cytokine IL-10 following DES implantation in patients with stable angina and to explore whether hypertensive status influenced this response. The findings reveal a biphasic IL-10 profile in which a significant elevation immediately post-procedure followed by a marked decline at 24 h, with levels falling below baseline. This pattern was observed in both hypertensive and normotensive patients, though hypertensive individuals exhibited a slightly greater initial IL-10 response.

The post-stenting rise in IL-10 aligns with previous reports that have identified this cytokine as an early compensatory mediator following vascular injury during PCI^9,10^. IL-10 is known to exert protective vascular effects by suppressing the expression of pro-inflammatory cytokines (e.g., IL-6, TNF-*α*), inhibiting endothelial activation, and modulating the activity of smooth muscle cells via NF-*κ*B inhibition^11,12^. Its transient upregulation after PCI likely reflects a host-driven attempt to counteract the mechanical and immunological insult of stent deployment.

Notably, the decline in IL-10 levels at 24 h suggests rapid attenuation of this anti-inflammatory defense. While we did not find a published study exactly mirroring this kinetics, our observation is congruent with the broader paradigm of a pro-/anti-inflammatory shift after vascular injury. For instance, Jakubiak et al. (2021) have emphasized that increased IL-6/IL-10 ratio may favor neointimal proliferation in in-stent restenosis^13^. Moreover, earlier human stent studies implicate IL-6 more consistently than IL-10 in restenosis, hinting that the anti-inflammatory IL-10 response may be less durable^14^. The transition in our data toward a higher IL-6/IL-10 ratio thus aligns with mechanistic expectations linking persistent low-grade inflammation to neointimal growth and late restenosis. The observed increase in the IL-6/IL-10 ratio at 24 h should be interpreted as an exploratory finding. While this ratio may provide insight into the net inflammatory milieu following PCI, the analysis was not predefined and is therefore hypothesis-generating. Confirmation of this pattern will require larger studies with predefined inflammatory endpoints.

Hypertension is a well-recognized risk factor for endothelial dysfunction and systemic inflammation. It has been linked to elevated basal IL-6 levels and increased vascular oxidative stress^15,16^. Although we did not observe statistically significant differences in IL-10 levels between hypertensive and normotensive patients at any time point, hypertensive individuals exhibited a more pronounced initial IL-10 surge. This could reflect heightened baseline inflammatory tone or increased sensitivity of vascular tissues to procedural injury. However, the rapid fall in IL-10 suggests that this response is short-lived and may be insufficient to counteract the sustained inflammatory stimulus in hypertensive patients. These observations should therefore be interpreted with caution and viewed as descriptive trends rather than evidence of a differential inflammatory response attributable to hypertension. The limited sample size and reduced statistical power preclude definitive conclusions regarding subgroup differences.

From a mechanistic perspective, IL-10 may protect against restenosis by inhibiting smooth muscle cell proliferation, matrix metalloproteinase activity, and macrophage activation—all processes implicated in stent failure^11^. The acute loss of IL-10 within 24 h, therefore, may represent a missed window of therapeutic opportunity. This raises the possibility that pharmacologic strategies to sustain IL-10 signalling in the early post-PCI period might improve vascular healing and reduce adverse remodelling, especially in high-risk groups such as hypertensive individuals.

This study offers several strengths that enhance the reliability of its findings. The use of well-defined sampling time point’s pre-procedural, immediately post-stenting, and 24 h post-procedure allowed for a detailed analysis of the temporal dynamics of IL-10 expression following drug-eluting stent implantation. By focusing on a homogeneous cohort of patients with stable angina undergoing elective PCI, the study minimized confounding effects related to acute coronary syndromes or systemic inflammatory conditions. Additionally, the stratification of patients based on hypertensive status provided clinically relevant insights into the interaction between vascular comorbidities and post-stenting inflammatory regulation.

This study has several important limitations. The small sample size and single-center design limit statistical power and generalizability, particularly for subgroup analyses. The observational design precludes causal inference. The short follow-up period restricts interpretation to early inflammatory changes and does not allow assessment of longer-term clinical outcomes such as restenosis. Additionally, the use of arterial sampling during PCI and venous sampling at 24 h introduces potential compartment-related variability in cytokine measurements. However, this approach reflects practical and ethical considerations during PCI and has been used in prior studies of acute inflammatory responses following coronary intervention. Finally, multiple comparisons were performed without formal adjustment, increasing the risk of type I error. These limitations underscore the exploratory nature of the findings. However, this study give an insight into the possible effects of IL-10 might have in patients with stable angina.

As arterial–venous cytokine gradients may exist, this difference in sampling compartments could have influenced absolute cytokine concentrations and partially confounded temporal comparisons. Finally, while cytokine measurements were performed, no mechanistic data were collected to link IL-10 dynamics to clinical or structural outcomes following stenting.

## Conclusion

In this study of patients with stable angina undergoing drug-eluting stent implantation, we observed a transient increase in circulating interleukin-10 levels immediately following the procedure, followed by a significant decline at 24 h. This pattern suggests a short-lived anti-inflammatory response to vascular injury, which may be insufficient to counterbalance the sustained pro-inflammatory stimuli associated with stenting. Although hypertensive patients demonstrated a more pronounced early rise in IL-10, no statistically significant differences were observed between hypertensive and normotensive groups. The concurrent increase in the IL-6/IL-10 ratio at 24 h highlights a shift toward a net pro-inflammatory state during the early post-procedural period. Taken together, these findings suggest a short-lived anti-inflammatory IL-10 response following drug-eluting stent implantation, followed by a relative pro-inflammatory shift within 24 h. While exploratory, these results highlight the importance of temporal cytokine regulation after PCI and provide a rationale for larger, prospective studies to evaluate the clinical relevance of IL-10 modulation.

## Conflicts of interest

The authors have declared no conflicts of interest.
